# Improving health service delivery in conflict-affected settings: Lessons from a nationwide strategic purchasing mechanism in Afghanistan

**DOI:** 10.7189/jogh.11.04049

**Published:** 2021-07-17

**Authors:** Christopher T Andersen, Habibullah Ahmadzai, Ahmad Wali Rasekh, Francisca A Akala, Trina Haque, Richard Johnson, Benjamin Loevinsohn, Ghulam D Sayed, Mickey Chopra

**Affiliations:** 1 The World Bank Group, Washington, D.C., USA; 2 The World Bank Group, Kabul, Afghanistan; 3 Ministry of Public Health, Kabul, Afghanistan; 4 Independent adviser on contracting and performance management, Cambridge, UK; 5 The Global Fund to Fight AIDS, Tuberculosis, and Malaria, Geneva, Switzerland

## Abstract

**Background:**

Due to ongoing insecurity, the government of Afghanistan delivers health care to the country’s population by contracting out service delivery to non-governmental organization service providers (SPs). In 2018, major changes to SP contracts were introduced, resulting in a new pay-for-performance service delivery model. This model, called “Sehatmandi”, pays SPs based on the volume of 11 key services they provide.

**Methods:**

A narrative review of Sehatmandi’s key features is presented, as well as lessons learned during implementation. Counterfactual comparisons of service delivery data for 10 payment-related service indicators are made. The first comparison is between the rate of change in the volume of services delivered from 2018 to 2019 (ie, the first year of Sehatmandi implementation) relative to the rate change from 2017 to 2018 (ie, prior to the program). The second comparison is between the rate of change in the volume of services delivered in provinces under the pay-for-performance mechanism relative to provinces which were not financed using pay-for-performance. Time trends in non-payment service indicators and service quality are also examined.

**Results:**

The increase in service volume in Sehatmandi provinces from 2018 to 2019 was higher than the increase from 2017 to 2018 for 8 out of 10 indicators. The median increase in the rate of change was 10 percentage points. Similar results were obtained when comparing pay-for-performance provinces to those not financed using pay-for-performance. Improvements were also observed for services that were not directly compensated by the pay-for-performance approach. Payment for service volume was not associated with reduced service quality. The narrative review suggests that the pay-for-performance system has stimulated more effective oversight of SPs by the government of Afghanistan and has incentivized innovative service delivery strategies by SPs. Sehatmandi may benefit from re-structuring its financial incentives to stimulate improved service quality and accelerate delivery of lagging services.

**Conclusions:**

The available evidence – though subject to some limitations – suggests that the introduction of a pay-for-performance system was associated with an expanded volume of service delivery in Afghanistan. This approach may be beneficial in other conflict-affected countries.

One-third of the global disease burden from HIV, tuberculosis and malaria, and some of the highest levels of maternal, newborn and child deaths are to be found in countries impacted by fragility, conflict and violence [1]. Despite persistent conflict and poverty, substantial improvements in health outcomes have occurred in Afghanistan since 2001, including a 50% decrease in child mortality and a 54% decrease in maternal mortality [2,3]. However, the context has remained fragile due to escalating violence since 2014. Over 10 000 civilians were killed or injured due to conflict each year from 2015-2019, and the proportion of the population falling below the national basic-needs poverty rate grew from 37% in 2012 to 55% in 2017 [4-6].

This challenging context required an innovative approach to health service delivery. The “System Enhancement for Health Action in Transition” project (SEHAT) supported delivery of primary and curative care services at the primary and secondary level between 2013-18 through a contracting out model [7]. Non-governmental organization service providers (SPs) were contracted by the Ministry of Public Health (MOPH) to deliver the MOPH’s Basic Package of Health Services (BPHS, which specifies essential primary care services to be delivered) and Essential Package of Hospital Services (EPHS, which specifies essential hospital services) [8,9]. This approach allowed for improved harmonization in service delivery activities among the many donor parties under the leadership of MOPH. SEHAT supported a substantial expansion in service delivery for many indicators, including a 45% increase in deliveries occurring in a health facility, a 176% increase in Caesarian sections, and a 44% increase in outpatient visits for child morbidity [10]. However, contraceptive use and vaccination rates stagnated, and levels for these indicators were particularly low in conflict-affected provinces [11].

A series of consultations examining the barriers to health care delivery and lessons learned from the five years of SEHAT implementation were initiated, culminating in a Presidential Summit on Health Care in Afghanistan in 2017, attended by President Ashraf Ghani. While the successes of the existing system were noted, it was identified that an improved system was needed which would clearly define success in service delivery and monitor the progress of SPs against that definition. The creation of a payment system linking payments to more specific performance indicators was critical to this process. A new pay-for-performance model – called Sehatmandi – was introduced in 2018 in response to these lessons, funded by the Afghanistan Reconstruction Trust Fund and the World Bank [12]. This paper describes the design of Sehatmandi and early results of implementation.

## METHODS

### Design of Sehatmandi

Under SEHAT, SPs were supervised by the Grants and Contract Management Unit of the Ministry of Public Health, whose main role is centered on procurement of SP services and contract compliance. With Sehatmandi, responsibility for oversight of SPs instead shifted to a broader group within the MOPH with a clear mandate for performance management. This group is coordinated by the Performance Management Office (PMO). SP performance is monitored by the PMO according to a prespecified set of criteria defined as part of the Standard Operating Procedures. Performance on these indicators is summarized into a score that is assessed every quarter at the sub-national level and every six months at national level. SPs that fail to obtain a satisfactory score are subject to sanctions and potentially a loss of contract. This change in management approach has been described as a shift from “contract management” to “performance management”. A comparison of the SEHAT and Sehatmandi models is available in **Table 1****.**

**Table 1:** Comparison of the design of the SEHAT and Sehatmandi health projects*

Design aspect	SEHAT	Sehatmandi
Party responsible for direct service delivery to population	Contracted non-governmental organization service providers	Contracted non-governmental organization service providers
Services delivered	BPHS and EPHS provided by different SPs	BPHS and EPHS **provided by same SP**
Funding mechanism	ARTF, IDA	ARTF, IDA, **GFF**
Process for selecting service providers	NGOs competitively bid on provincial-level contracts to deliver BPHS or EPHS services. Bidders submit lump-sum financial proposals. Contracts awarded based on the combination of technical and financial proposal scores.	NGOs competitively bid on provincial-level contracts to deliver BPHS **and** EPHS services. Bidders submit lump-sum financial proposals **to cover overheads accounting for expected pay-for-performance revenues. MOPH sets “tariffs” for 11 key services.** Contracts awarded based on the combination of technical and financial proposal scores.
Party responsible for oversight of service providers	Grants and Contracts Management Unit	**Performance Management Office**
Payment of service providers	Service providers paid lump sum installments divided evenly across the contract period.	Service providers paid lump sum installments divided evenly across the contract period, **and pay-for-performance installments initially every six months and later every three months based on independently-verified service volume reports.**
Metrics for monitoring service providers	Service providers paid lump-sum amount (based on bid price) if performance is judged to be “adequate” by third party monitor. 20% of payments were linked with achievement of a pre-defined percent of key services.	**Service providers paid based on service delivery volume (contingent on acceptable quality of care, accuracy of reporting, timeliness of staff payments, and timeliness of reporting). Consequences for poor performance are pre-specified.**
		***The criteria used to evaluate service providers include: volume of service delivery relative to pre-specified minimums, accuracy of HMIS reporting, adherence to a “minimum set of standards” of service quality and a composite score of broader service quality as assessed by a third party monitor, quality of care indicators as assessed by MOPH technical departments, timeliness of reporting to MOPH, and timeliness of salary payments to staff.***
External monitoring	A third-party monitor conducts audits of service volume reports each six months and a survey of health facility quality every 1-2 y.	A third-party monitor conducts audits of service volume reports each six months and a survey of health facility quality every 1-2 y.

ARTF – Afghanistan Reconstruction Trust Fund, BPHS – Basic Package of Health Services, EPHS – Essential Package of Hospital Services, GFF – Global Financing Facility, HMIS – Health Management Information System, IDA – International Development Association, MOPH – Ministry of Public Health, NGO, non–governmental organization

*Differences between projects are highlighted in bold.

Three alternative models of strategic purchasing were considered for the design of Sehatmandi (**Table 2**). All models relied on the introduction of SP payments being linked to service delivery volume and service-specific tariffs. Under one model, tariffs would be set by MOPH and SP bidders would compete on the cost of a lump sum portion. In a second model, tariffs would be proposed by the SP bidders, with the lump sum portion capped at 30% of the total contract price. The final model option allocated a lump sum associated with a baseline level of services, and services delivered beyond that threshold would be paid based on the tariff. Considering the relative advantages and disadvantages, the first model was used. Under this model, the base tariff was set for each service according to the findings from either an external costing study (for eight services) or an internal MOPH evaluation (for three services) [13]. Tariffs were then adjusted for each province to account for differences in the cost of service delivery by dividing the cost per capita of the SEHAT project contracts in each province by the cost per capita in the external costing study.

**Table 2:** Comparison of strategic purchasing options for health care system in Afghanistan

Option	Description	Advantages	Disadvantages
1	• Fixed tariffs defined centrally by MOPH	• No requirement for MOPH to apply ‘judgment’ in evaluating tariffs proposed by providers	• Service providers may argue tariffs are too low if they fall short on delivering services
		• Use of multipliers ensures equity	
	• Use of multipliers to adjust tariff to the cost of service delivery in different provinces	• Service providers can account for tariffs they believe are too low by increasing the lump sum bid amount	• Providers may need technical assistance with business planning due to minimal experience with costing by service
	• Pre-defined and transparently applied option for changes to tariffs for all provinces	• Strengthens the link between the cost of operational delivery and the delivery of health outcome	• Potential resistance to different tariffs for the same services in different provinces
	• Service providers will compete on price based on a lump sum amount to cover fixed costs	• Incentivizes increased coverage	• Potential inequity as providers target easy to reach groups and avoid those for whom marginal cost is higher
2	• Tariffs proposed by service provider bidders	• Tariffs potentially more realistic for a given province	• Risk of gaming the tariff structure by setting higher amounts of services that are easier to deliver
	• Lump sum capped at 30% of total bid price	• The cap on the lump sum will help ensure most of the payments will be linked with performance	• May be difficult for service providers to calculate realistic tariffs
	• Total contract value and tariffs are not constrained up front, however lump sum capped at 30% of total bid price	• Incentivizes increased coverage	• Increased inequity as providers target easy to reach groups and marginal cost for hard to reach more than tariff
3	• The baseline level of services is covered by a lump sum bid	• Lower financial risk for implementers due to smaller proportion of revenue linked to performance	• Potentially insufficient financial incentive for exceeding the baseline level of services
	• Tariffs are paid for services delivered beyond the baseline level	• Incentivizes increased coverage	

MOPH – Ministry of Public Health

Competitive contract bids submitted by potential SPs comprised a technical component (weighted at 70%) and a cost component (weighted at 30%). Under the cost component, SPs bid competitively only on the lump sum proportion of the contract. The objective of this approach was to give SPs greater control over their delivery models, to encourage them to consider the relationship between their expenditure and their performance, and to incentivize them to find efficient, locally responsive ways to deliver more services. Contracts were awarded to cover both BPHS and EPHS services on a per-province basis, for 31 out of 34 provinces (in the remaining three provinces, MOPH directly manages services).

After contracts were awarded, the lump sum payments to SPs were divided equally into installments which are disbursed regularly throughout the contract period. Pay-for-performance payments are calculated based on the number of services reported through the Health Management Information System (HMIS) and verified by a third-party monitor (TPM; see **Table 3** for an example of how payments are calculated). HMIS reports are submitted on a monthly basis by each health facility, aggregated at the provincial level, and then submitted to the central MOPH HMIS team on a quarterly basis (details on the HMIS reporting system are available in Appendix S1 in the **Online Supplementary Document**). Every six months, the TPM takes a sample of health facilities in each province to verify reported service delivery data (see Appendix S2 in the **Online Supplementary Document** for information on HMIS data verification and accuracy). The count of services reported to HMIS is compared to the registers for all 11 pay-for-performance services, and then a sample of those services are further audited by contacting the patients to confirm whether they in fact received the services. This verification is then used to revise any payments to the SPs which have already been made on the basis of their self-reporting. In order to ensure spending on the Sehatmandi project did not outstrip the available resources, a cap was set for each service in each province for the maximum number of services that could be reimbursed. Caps were set based on historical trend data from household surveys and HMIS reports.

**Table 3:** Example of service provider payment calculation for pay-for-performance services using national base tariff

Indicator	A. Delivery volume during payment period (reported by service provider)	B. Proportion of services verified (audit by third-party monitor)	C. Number of verified services (A*B)	D. Per-service tariff (USD)	E. Payment amount (USD; C*D)
Antenatal visits	29 089	92%	26 797	2.90	77 711.59
Caesarian sections	1 309	90%	1 174	192.60	226 112.40
Couple-years of protection	4 032	83%	3 355	3.90	13 084.50
Growth monitoring	122 179	77%	93 545	1.10	102 899.57
Institutional deliveries	8 134	100%	8 134	13.80	112 49.20
Major surgeries	803	100%	803	125.60	100 856.80
Outpatient cisits (children <5 years)	348 180	86%	299 313	1.50	448 969.59
Pentavalent dose 3 vaccinations	22 244	93%	20 618	1.70	35 050.81
Postnatal visits	23 148	85%	19 776	4.30	85 037.18
Tetanus 2+vaccinations	79 964	89%	71 440	1.70	121 447.97
Tuberculosis cases treated	208	96%	201	17.60	3530.06
				**Grand total**	**1** **326** **949.67**

USD – United States dollars

## RESULTS FROM FIRST YEAR OF IMPLEMENTATION

**Figure 1:**
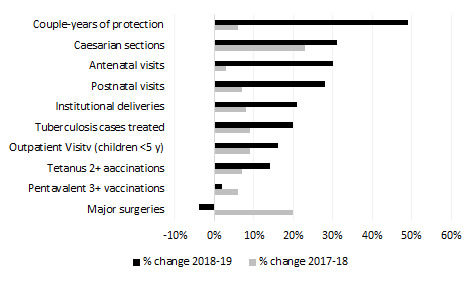
Improvements in pay-for-performance services before and after the introduction of Sehatmandi among Sehatmandi service providers in contracted-out provinces.

Trends in service delivery were evaluated using HMIS data from 2017-2019 (see Appendix S3 in the **Online Supplementary Document** for a description of the evaluation methodology). The volume of services delivered in 2019 (under Sehatmandi) compared to 2018 (under SEHAT) increased for 9 out of 10 payment indicators with data for both years (data for the 11th payment indicator, growth monitoring, was only collected in 2019; **Table 4**), although the growth in service volume varied widely (-4% to +49%). Furthermore, the rate of change in 2018-19 was higher than the rate of increase from 2017-18 for 8 out of 10 services (**Figure 1****,** **Table 4**). A second comparison was made between Sehatmandi providers in the 31 contracted-out provinces compared to the three provinces where MOPH directly manages services. Since MOPH-managed providers were not receiving pay-for-performance payments, then it is plausible that differences in the rate of change from 2018-19 between these two types of SPs could be attributable to the introduction of the Sehatmandi model. The rate of change of service volume was higher in Sehatmandi provinces relative to MOPH-managed provinces for 8 out of 10 services (**Table 5**).

**Table 4:** Improvements in pay-for-performance services before and after the introduction of Sehatmandi among Sehatmandi service providers in contracted-out provinces*

Indicator	2017	2018	2019	% change 2017-18	% change 2018-19	Difference in rate of change
Couple-years of protection	267 127	284 091	423 320	6%	49%	43%
Antenatal visits	2 127 785	2 183 546	2 831 830	3%	30%	27%
Postnatal visits	1 197 476	1 277 062	1 636 018	7%	28%	21%
Institutional deliveries	516 916	559 448	674 372	8%	21%	12%
Tuberculosis cases treated	12 403	13 497	16 212	9%	20%	11%
Caesarian sections	13 373	16 441	21 569	23%	31%	8%
Outpatient visits (children <5 years)	11 575 334	12 564 387	14 518 430	9%	16%	7%
Tetanus 2+vaccinations	2 927 625	3 143 672	3 573 828	7%	14%	6%
Pentavalent dose 3 vaccinations	1 043 829	1 111 086	1 133 145	6%	2%	-4%
Major surgeries	34 408	41 292	39 784	20%	-4%	-24%
Growth monitoring	n/a	n/a	5542323	n/a	n/a	n/a
**Median change**				**8%**	**21%**	**10%**

n/a – not applicable

*Health management information system data submitted by service providers.

**Table 5:** Improvements in pay-for-performance services comparing Sehatmandi providers in 31 contracted-out provinces and MOPH-managed facilities in 3 provinces*

	Contracted-out Sehatmandi providers			MOPH-managed providers			Difference in change
**Indicator**	**2018**	**2019**	**% change**	**2018**	**2019**	**% change**	
Couple-years of protection	284 091	423 320	49%	21 548	24 895	16%	33%
Antenatal Visits	2 183 546	2 831 830	30%	133 469	141 568	6%	24%
Postnatal visits	1 277 062	1 636 018	28%	68 702	75 675	10%	18%
Outpatient visits (children <5 years)	12 564 387	14 518 430	16%	656 153	655 070	0%	16%
Tetanus 2+vaccinations	3 143 672	3 573 828	14%	134 379	137 060	2%	12%
Institutional deliveries	559 448	674 372	21%	26 898	30 524	13%	7%
Caesarian sections	16 441	21 569	31%	792	1 012	28%	3%
Pentavalent dose 3 vaccinations	1 111 086	1 133 145	2%	48 495	47 878	-1%	3%
Tuberculosis cases treated	13 497	16 212	20%	673	832	24%	-4%
Major surgeries	41 292	39 784	-4%	2 554	2 651	4%	-7%
Growth monitoring	n/a	5 542 323	n/a	n/a	111 425	n/a	n/a
**Median change**			**21%**			**8%**	**10%**

n/a – not applicable

*Health management information system data submitted by service providers.

The risk of SPs inflating reported service count data are mitigated by high penalties for inaccurate reporting backed by independent, random audits. In the first year of implementation, the TPM completed all data collection activities for both HMIS verifications and health service quality despite security challenges. HMIS data accuracy (as assessed by an “HMIS Verification Index”, which indicates the proportion of services reported by SPs to HMIS that are subsequently verified) during Sehatmandi’s first 9 months of operation found that 5 services had accuracy greater than 90%, and the remaining 4 services had accuracy above 75%. Comparisons of HMIS data in the pre- and post-Sehatmandi period also seem to be relatively unaffected by misreporting (see Table S1 in Appendix S2 in the **Online Supplementary Document**).

**Figure 2:**
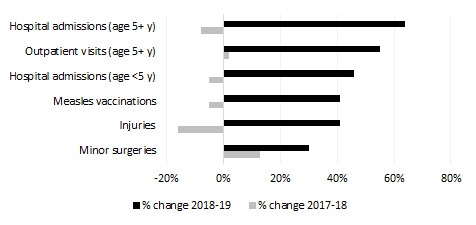
Improvements in non pay-for-performance services among Sehatmandi providers in contracted-out provinces before and after the introduction of Sehatmandi.

The introduction of pay-for-performance contracts did not appear to negatively impact the volume of non-payment indicator services delivered by the same SPs. Six non-payment services (selected to represent service delivery that is not financially incentivized by the 11 payment indicators) had a greater rate of change in the post-Sehatmandi period compared to pre-Sehatmandi (**Figure 2****,** **Table 6**). This evidence suggests that the introduction of payment indicators has not harmed non-payment indicators, and in fact may have benefitted them.

**Table 6:** Improvements in non pay-for-performance services among Sehatmandi providers in contracted-out provinces before and after the introduction of Sehatmandi*

Indicator	2017	2018	2019	% change 2017-18	% change 2018-19	Difference in rate of change
Hospital admissions (ages 5 years or more)	1 775 603	1 636 834	2 679 228	-8%	64%	71%
Injuries	236 737	199 404	280 812	-16%	41%	57%
Outpatient visits (ages 5 years or more)	106 673 128	109 202 688	169 613 520	2%	55%	53%
Hospital admissions (children <5 years)	419 476	397 936	580 448	-5%	46%	51%
Measles vaccinations	4 709 546	4 457 282	6 273 164	-5%	41%	46%
Minor surgeries	794 383	901 554	1 175 404	13%	30%	17%

*Health management information system data submitted by service providers.

Finally, financial incentives to increase service volume does not appear to have led to a significant deterioration in quality of services (as measured by the Balanced Scorecard assessment [BSC]). The BSC measures indicators of service quality according to 5 domains (including client satisfaction, human resources, physical capacity, quality of provider interactions, and provider management) and has been consistently administered by a TPM across all SPs since 2004. These indicators are then compiled into a total score scaled from 1-100. The total score in 2018 was 59.3 and in 2020 was 58.5 [14,15]. Since scores between 2011 and 2017 had varied between 55.0 and 63.5, the findings suggest that there was no adverse effect on service quality.

It is important to note that the non-randomized implementation of the Sehatmandi program prevents an unqualified causal interpretation of the associations presented here. In theory, there could have been other changes that were correlated with the introduction of Sehatmandi and instead resulted in increased service delivery volume. Furthermore, little data are available on the accuracy of the HMIS reports prior to the introduction of Sehatmandi; yet data from 2019 shows accuracy to be reasonably high for most indicators. Despite these caveats, the consistency and magnitude of the observed association make a plausible case for the beneficial impact of Sehatmandi. Increases in service delivery volume were consistently seen when comparing pre- vs post-Sehatmandi trends, SP- vs MOPH-managed provinces, and for both payment and non-payment services. Furthermore, the magnitude of association was considerable (median increase in service volume of 10 percentage points). Taken together, the authors believe these results are worth strong consideration by policy makers globally.

### Key lessons

The introduction of the pay-for-performance model has made it clear that a performance-based system of payments and a pre-specified set of metrics for supervision is feasible even within a conflict-affected setting. Indeed, it may have a number of advantages. Whereas decision-making had often been made previously based on anecdotal evidence and political considerations, the Standard Operating Procedures have helped to lessen these concerns. Since SPs know what criteria they will be evaluated against, they can concentrate their efforts on achieving those objectives. With payments linked to performance rather than simply budget reimbursement, SPs have the flexibility (and incentive) to adapt resources in response to the fragile, changing context.

The introduction of performance-based contracts has also stimulated increased learning opportunities for SPs to improve service delivery. The establishment of the PMO has resulted in increased supervision (all provinces were visited by PMO staff in the first six months of Sehatmandi) with opportunities to troubleshoot issues. MOPH has also convened SPs to engage in learning sessions where they discuss their experiences and learn from other providers. SPs are motivated to participate in these activities because there is a financial reward to be gained if their newly applied knowledge results in expanded service delivery.

SPs clearly modified their behavior in response to the pay-for-performance incentive. In fact, many SPs began incorporating pay-for-performance components into staff salaries. This has reportedly served to help reduce absenteeism. However, it is also important to note that health facility staff need to have their salaries protected from impacts on service volume that are outside of their control, such as interference by anti-government armed groups and mismanagement by SPs. As a result, the MOPH has mandated that only 20% of staff salaries may be conditioned on service delivery volume.

In order to further increase service delivery, SPs have developed numerous innovative strategies. Some have addressed supply-side constraints, such as extending the opening hours for clinics along with shift-working for staff. Others have sought to increase demand; some examples include paying bonuses to community health workers who refer patients for services and providing transport for women to and from Caesarian section operations. The flexibility of the performance-based payment system was meant to incentivize exactly this kind of creativity.

One challenge in the implementation of the project is ensuring adherence to the Standard Operating Procedures (SOPs) by MOPH staff. The SOPs are comprehensive in their coverage of performance issues, so adequate training is required to ensure that both members of the PMO and other departments within MOPH are aware of the protocols that should be applied to a given situation. Consistent and fair application of the SOPs is expected to improve trust and an effective working relationship between MOPH and SPs. It is therefore important for there to be oversight of MOPH itself to ensure it is adhering to the SOPs. MOPH and financing partners have agreed to introduce a new layer of performance reporting and to begin holding regular reviews to provide this oversight.

As seen in **Figure 1**, some services have not grown substantially after the introduction of Sehatmandi. For some of these services, such as vaccinations, it is possible that the tariffs are not high enough to cover the cost of service delivery or to incentivize additional investment. On the other hand, for certain services, service volumes have exceeded the payment caps set in the contract. Since demand is clearly higher than the caps for these services, it is important not to create a perverse incentive for SPs to constrain care. Tariffs must be iteratively reviewed to ensure adequate performance for all services.

While the results of the BSC indicate there was no negative impact of Sehatmandi on service quality, it also shows that service quality has not improved. A critical next step will be to drive enhancements in service quality. While SPs are penalized if service quality falls below a set threshold, the current financial structure does not incentivize quality to increase.

## CONCLUSION

The available data – while subject to limitations – suggest that the Sehatmandi strategic purchasing model is associated with bolstered health service delivery in Afghanistan, despite high levels of poverty and conflict. The approach described here additionally relies on a performance management approach stewarded by MOPH with a well-defined SOP to ensure transparency and accountability. It is also driven by robust data collection and the use of data in decision-making. Other conflict-affected settings may wish to consider implementing a pay-for-performance model based on these findings.

## Additional material

Online Supplementary Document
