# Supplementary material for: Improving health service delivery in conflict-affected settings: Lessons from a nationwide strategic purchasing mechanism in Afghanistan
Source: J Glob Health. 2021 Jul 17;11:04049. doi: 10.7189/jogh.11.04049 (PMC8285764; doi:10.7189/jogh.11.04049)
Supplement: Online Supplementary Document [file jogh-11-04049-s001.pdf]

Appendix S1. Data collection via the HMIS

The Ministry of Public Health in Afghanistan has maintained a Health Management Information System (HMIS) since 2004. On a monthly basis, every publicly financed health facility in Afghanistan tabulates the number of services they provide for key indicators using standardized reporting forms. These reports are submitted by facilities to the Ministry of Public Health and recorded in an electronic database. As of 2019, there were 1,012 sub-health centers, 878 basic health centers, 433 comprehensive health centers, and 85 district hospitals represented in the database. The indicators included in this study are as follows:

1. Antenatal visits: Total number of antenatal care visits provided to pregnant women.
2. Postnatal visits: Total number of postnatal care visits provided to recently delivered women.
3. Pentavalent dose 3 vaccinations: Total number of children who were given their third dose of the pentavalent vaccine.
4. Institutional deliveries: Total number of women who delivered their baby at an appropriate health care facility.
5. Tetanus 2+ vaccinations: Total number of vaccines administered to pregnant or non-pregnant women which were the client's 2nd dose or more.
6. Couple-years of protection: The total number of years of contraception provided to clients via a variety of modern contraceptive methods. The contraceptive methods (and years of contraception for each) are: one cycle of oral contraceptives (0.07 years), injectable contraceptives (0.25 years), intrauterine devices (3.5 years), pack of 12 condoms (0.08 years), implants (3.8 years), and permanent contraception methods (12.5 years). The number of years of protection associated with each method is per recommendations by the US Agency for International Development.
7. Outpatient Visits (Children <5 y): Total number of outpatient visits for children under 5 years.
8. Caesarian sections: Total number of Caesarian sections performed during delivery.
9. Tuberculosis cases treated: Total number of sputum-positive tuberculosis cases that have completed treatment and were confirmed as sputum-negative.
10. Major surgeries: Total number of surgeries classified as "major" performed by the health facilities.
11. Growth monitoring: Total number of visits for children under 2 years where growth monitoring and nutritional counseling is performed.

Appendix S2. Verification of HMIS data

Since 2015, a subset of indicators submitted to the HMIS has been validated by a third-party monitor. The specific services verified by the third-party monitor has varied over time, according to the Ministry of Public Health's needs; however, the sampling methodology has remained similar. A subset of approximately 25% of all functional health facilities is selected for monitoring every 6 months (from 2017-2019, the number of facilities surveyed in each round ranged from 406-534). First, a Consistency Index for each facility is calculated by dividing the number of services recorded in the health facility register by the number of services submitted to the HMIS. Second, 4-6 clients listed in the register are contacted to confirm if they received the service recorded. An Accuracy Index is then calculated by dividing the number of clients who were located and who confirmed receipt of service by the number of clients contacted. Finally, an HMIS Verification Index is calculated by multiplying together the Consistency Index and Accuracy Index, and then multiplied by 100. The HMIS Verification Index can be interpreted as the percentage of services submitted to the HMIS that were actually provided.

The mean HMIS Verification Index of all facilities for relevant indicators is presented in Table S1.

Table S1. Mean HMIS Verification Index for relevant indicators assessed at publicly financed health facilities in Afghanistan*

	2017	2018	Jan-Sep 2019
		(Score out of 100)	
Antenatal Visits	75.8	90.2	90.7
Postnatal Visits	79.3	85.9	86.2
Pentavalent Dose 3 Vaccinations	84.5		93.3
Institutional Deliveries			90.0
Tetanus 2+ Vaccinations			86.5
Tuberculosis Cases Treated			95.1
Growth Monitoring			80.2
Outpatient Visits (Children <5 y)			75.8
Caesarian Sections			91.9

*The following types of facilities are included in this data: sub-health centers, basic health centers, comprehensive health centers, and district hospitals.

Appendix S3. Methodology for counterfactual comparisons

For Tables 4-6, the sum of services for all health facilities for the years 2017, 2018, and 2019 were calculated. The percent change in the total number of services provided was calculated for 2017-2018 (pre-Sehatmandi), and 2018-2019 (post-Sehatmandi). For Tables 4 and 6, the difference in the rate of change between these two periods was then calculated. The difference in the rate of change was selected as the quantity of interest, as opposed to the difference in the volume of services, because service volume was already expanding prior to the introduction of Sehatmandi. In Table 5, the rate of change from 2018-2019 is compared between contracted-out Sehatmandi providers and MOPH-managed providers. This comparison is relevant because MOPH-managed providers were not paid based on the volume of service delivery. A comparison of the rates of change in Table 5 is informative because it accounts for the fact that fewer health facilities are managed by the MOPH, and therefore the total volume of services delivered is lower.

The exclusion of confidence intervals and statistical tests from this analysis is intentional. HMIS data is available for all services provided in Afghanistan (not just a sample of services). Therefore, the principle of imprecision due to sampling error (which is the basis for confidence intervals and statistical tests) does not apply. For a reference of this approach, see: Kaplan D. Statistical Modeling: A Fresh Approach [section 5.7]. Second edition (July 2017). Accessible at https://dtkaplan.github.io/SM2-bookdown/
